# Evolution of visual art with dopaminergic therapy

**DOI:** 10.1186/s40734-016-0034-y

**Published:** 2016-03-21

**Authors:** Ruth H. Walker

**Affiliations:** Department of Neurology, James J. Peters Veterans Affairs Medical Center, 130 W. Kingsbridge Road, Bronx, NY 10468 USA; Department of Neurology, Mount Sinai School of Medicine, New York City, NY USA

**Keywords:** Parkinson’s disease, Art, Attention, Dopamine

## Abstract

A patient with right-side-predominant Parkinson’s disease presented visual artwork which improved in resemblance to the model which he was copying with increasing doses of levodopa. I propose that increased dopaminergic replacement resulted in improved attention to detail, mediated by circuitry in the left hemisphere.

## Background

The emergence or evolution of artwork with the progression of neurodegenerative diseases has been reported in a number of cases, especially frontotemporal dementia [1], but also in Parkinson’s disease (PD) [2–4]. Here I report a man whose visual art developed with increased detail and realism over a few months as his dopaminergic therapy increased. This work was produced at an art class in which students copied a specific picture, and the patient’s wife’s work at the same art class was available as a useful comparison. There was no evidence of behavioral compulsion. I propose that the increase in visual details was attributable to an increase in attention with the increase in dopaminergic stimulation, rather than an increase in creativity.

## Case presentation

**Fig. 1:**
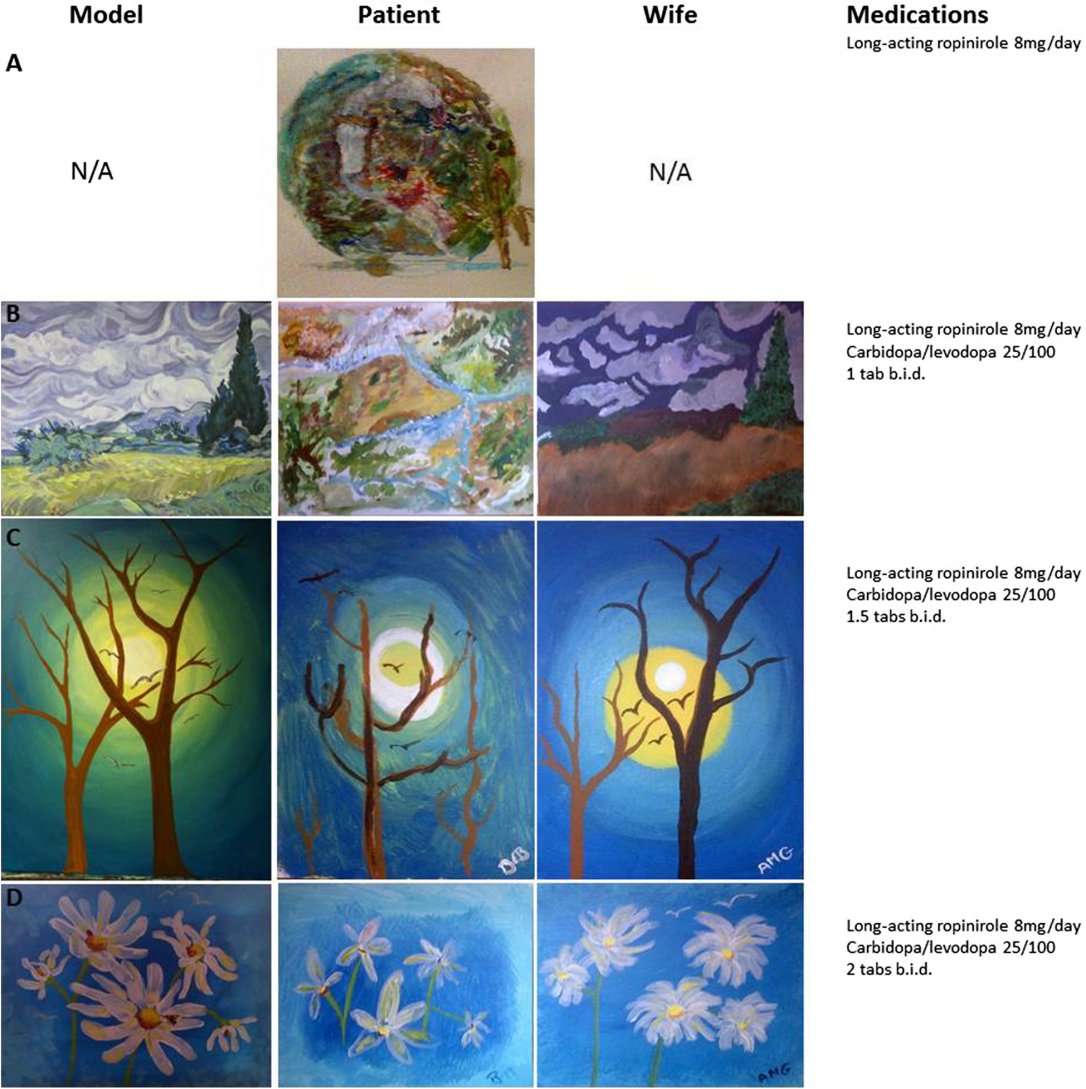
The patient’s artwork ordered temporally from top to bottom (*center panels*), along with the model provided by the art teacher (*left panels*), his wife’s work at the same art class (*right panels*), and the dose of dopaminergic medication that he was taken at the time. The patient’s image in row A was produced in the class as a copy of a model, but his wife copied a different painting and they cannot recall what he was meant to be copying, and thus equivalent images are not available

A 68-year old right-handed former high school mathematics teacher was referred for evaluation of PD. He was being treated with long-acting ropinirole 8 mg/day and was obviously undertreated with significant bradykinesia, rest tremor, and rigidity, all of which were more marked on the right than the left. Carbidopa/levo-dopa 25/100 1 tablet b.i.d was started. Over the next 6 months this was increased to 1.5 tabs b.i.d, and then 2 tablets b.i.d., in addition to an unchanged dose of long-acting ropinirole, with a significant reduction in bradykinesia and improvement in functioning. (He preferred this dosing schedule for convenience.) The patient had previously reported an interest in drawing, but had never pursued this, despite his wife providing materials for him. He had never engaged in any other creative activity, such as creative writing or music. His wife urged him to join her at a regular art class where students copied a work presented by the class instructor. His wife reported a progressive increase over time in the level of detail and similarity to the copied work (Fig. 1). Interestingly, in the 3^rd^ image in the series (row C), the patient painted a mirror image of the model painting. He did not produce art outside the classes, and there were no signs of any compulsive behaviors or other neuropsychiatric complaints.

The patient’s wife also noted that when he was initially diagnosed with PD he was not able to perform simple arithmetic or solve mathematical word problems. These functions improved as his dopaminergic therapy was increased, also suggesting that he had improved ability with tasks requiring attention, as discussed below.

The patient has given his consent for this case report to be published.

## Conclusions

The evolution of the patient’s work is striking, with increased similarity between the model and the produced work with medication increase. It is not clear that this was due to an increase in the patient’s creativity, even though an increase in creativity in PD, especially with the use of dopaminergic medications, appears to be a significant phenomenon and has recently been discussed in detail [2]. A number of cases have been reported in which patients with PD started producing artwork, in some cases in prodigious quantities, as the disease progressed. This has been seen not only with visual arts, but also with writing of various forms [2]. It is postulated that the combination of PD and therapy with dopaminergic agents is required for this creativity to emerge. In certain situations the drive to produce artwork appears to be part of a behavioral compulsion [3, 4], although obviously with a more concrete product, unlike with punding [5]. However, this does not apply to the present case, nor to many others [6], in which there is a specific goal of the activity.

It is possible that the improved resemblance of the patient’s image to the model could be explained by an improvement in his motor abilities with treatment, however, I believe that this is unlikely, as the most striking change is the improvement in the similarity of the content to the model. He clearly was able to paint straight lines in row B (center panel), however, the product bore only a general resemblance to his wife’s work (a landscape with hills and trees; right panel), as compared with his later pieces. Nor is it likely that there was a significant improvement in his technical skill with successive art classes – these classes only occurred every few months, and he was not painting at home in between.

One attractive hypothesis is that the improvement was related to an increase in visual attention with the increase in dopaminergic medication. Visual attention is known to be impaired in PD and may be improved by dopaminergic medications [7, 8]. While it has been broadly conceptualized that artistic ability is localized to the right hemisphere, it is now apparent that both hemispheres contribute to different aspects to the production of artistic work, and this may be a factor in the present case. It may be relevant that the patient’s symptoms were more marked on the right side of the body, and that attention to visual detail is localized to the left hemisphere, specifically the parietal cortex. Processing of local visual signals has been reported to be more impaired in patients with right-sided PD [9]. Thus it is possible that this function was impaired by dopamine deficiency, and was improved by replacement. Another mechanism that may have contributed is improved motor attention, which also localizes to the left parietal cortex [10]. It was also reported that the patient’s ability to perform mathematics improved as his dopaminergic medication was increased, suggesting that cognitive attention was the critical function. Serial neuropsychological evaluations would have been informative, however, these were not performed.

In general, it appears that dopaminergic agonists are typically responsible for behavioral changes in PD, thus it is of note that in the present case the dose of dopamine agonist was constant, while the levo-dopa dose was increased. This may suggest that what is reported here is not due to a change in behavior, but rather is due to improved dopaminergic functioning mediated by other cognitive mechanisms.

## Consent

Written informed consent was obtained from the patient for publication of this case report and any accompanying images. A copy of the written consent is available for review by the Editor-in-Chief of this journal.

## Abbreviations

PD: Parkinson’s disease
